# Altered Default Mode Network on Resting-State fMRI in Children with Infantile Spasms

**DOI:** 10.3389/fneur.2017.00209

**Published:** 2017-05-19

**Authors:** Ya Wang, Yongxin Li, Huirong Wang, Yanjun Chen, Wenhua Huang

**Affiliations:** ^1^Institute of Human Anatomy, Southern Medical University, Guangzhou, China; ^2^Electromechanic Engineering College, Guangdong Engineering Polytechnic, Guangzhou, China

**Keywords:** epilepsy, infantile spasm, functional connectivity, default mode network, resting-state fMRI, amplitude of low-frequency fluctuation, temporal lobe

## Abstract

Infantile spasms (IS) syndrome is an age-dependent epileptic encephalopathy, which occurs in children characterized by spasms, impaired consciousness, and hypsarrhythmia. Abnormalities in default mode network (DMN) might contribute to the loss of consciousness during seizures and cognitive deficits in children with IS. The purpose of the present study was to investigate the changes in DMN with functional connectivity (FC) and amplitude of low-frequency fluctuation (ALFF), the two methods to discover the potential neuronal underpinnings of IS. The consistency of the two calculate methods of DMN abnormalities in IS patients was also our main focus. To avoid the disturbance of interictal epileptic discharge, our testing was performed within the interictal durations without epileptic discharges. Resting-state fMRI data were collected from 13 patients with IS and 35 sex- and age-matched healthy controls. FC analysis with seed in posterior cingulate cortex (PCC) was used to compare the differences between two groups. We chose PCC as the seed region because PCC is the only node in the DMN that directly interacts with virtually all other nodes according to previous studies. Furthermore, the ALFF values within the DMN were also calculated and compared between the two groups. The FC results showed that IS patients exhibited markedly reduced connectivity between posterior seed region and other areas within DMN. In addition, part of the brain areas within the DMN showing significant difference of FC had significantly lower ALFF signal in the patient group than that in the healthy controls. The observed disruption in DMN through the two methods showed that the coherence of brain signal fluctuation in DMN during rest was broken in IS children. Neuronal functional impairment or altered integration in DMN would be one neuroimaging characteristic, which might help us to understand the underlying neural mechanism of IS. Further studies are needed to determine whether the disturbed FC and ALFF observed in the DMN are related to cognitive performance in IS patients.

## Introduction

Infantile spasms (IS) syndrome, or West syndrome, one kind of intractable epileptic encephalopathy, is characterized by early-onset flexion–extension motor spasms, mental retardation or regression, developmental delay, and a pathognomonic electroencephalography (EEG) pattern of hypsarrhythmia, which consists of a chaotic and disorganized activity with asynchronous large amplitude slow waves mixed with single, multifocal spikes and sharp waves followed by attenuation (1–3). Because epileptic seizure itself causes and aggravates behavioral and developmental performances, neurocognitive outcome of IS is consequently poor (4, 5).

The mechanism of seizure and neurocognitive deterioration in IS is complex. Previous studies from Chugani and colleagues have shown that glucose metabolism positron emission tomography scanning could localize abnormal lesions to help surgical decision-making, especially in patients with normal MRI and bilateral EEG abnormalities in epileptic spasms patients with or without IS (6). A previous study about EEG has shown that IS patients have significant abnormalities in coherence and spectral power (3). But the potential neuronal underpinnings of impaired cognition and functional brain reorganization are not fully elucidated. The functional brain networks that are involved in cognitive processing can be identified through fMRI. Brain regions with negative blood oxygen level-dependent (BOLD) activity during cognitive processing are defined as default mode network (DMN) in which the regions are functionally connected with each other during rest when spontaneous fluctuations of the BOLD time course occur (7). DMN regions are considered to be related to abnormal functional integration and ictal unconsciousness in generalized epilepsy, which encompass the anterior and posterior cortical midline structure, lateral parietal cortex, temporal cortices, and hippocampus formation (7, 8). Therefore, we intended to measure the functional changes in DMN to interpret the neuronal underpinnings with resting-state fMRI.

Functional connectivity (FC) analysis, relying on regions of interest (ROIs), could detect interregional temporal correlations based on BOLD signal oscillations (9). Then it could reflect the pathways of information transmission between brain regions. The key point about the occurrence of epilepsy might be related to the changed transmitted pathways in the posterior of the brain. Therefore, we employed FC to explore the neuronal mechanism. Abnormal interregional FC in DMN usually occurs in Alzheimer’s disease (10), Parkinson’s disease (11), and depression (12). Altered FC within the DMN also has been found in several types of epilepsy, including idiopathic generalized epilepsy (13, 14), temporal lobe epilepsy (15–17), and absence epilepsy (18). But there are few studies that are focusing on the alteration of FC in DMN in IS patients. Therefore, we intend to explore altered regions in IS patients with FC.

Amplitude of low-frequency fluctuation (ALFF), an effective data-driven analysis technique based on fMRI, can directly measure the ALFF of the BOLD signal in each region. It is usually used to explore brain functions of both healthy subjects (19) and clinical patients (20). Up to now, it has been increasingly used for investigating the functional alteration in many kinds of epilepsy (21–23). For example, some studies have showed that aberrant ALFF is located in DMN regions in patients with temporal lobe epilepsy (21, 24). But few studies focus on this method in IS patients. So in the present study, we focus on the whole alteration of amplitude in patients with IS using the ALFF method.

Consequently, in our present study, we utilized the FC and ALFF methods to analyze the fMRI data from IS patients and healthy controls. We chose the two different methods to characterize the differences between the two groups and to investigate the consistency under different methods. On the basis of our findings, we try to explain how the IS affect the brain function and which regions are potentially associated with impaired cognition. On the basis of previous studies of epilepsy (17, 24), we hypothesized that the coherence of brain signal fluctuations in DMN may have been broken in the children with IS.

## Materials and Methods

### Subjects

A total of 13 right-handed patients (3 females; mean 2.9 years) with IS and 35 sex- and age-matched controls (13 females; mean 2.5 years) were recruited from the Shenzhen Children’s Hospital, Guangdong, China. Two of them were cryptogenic. One or two antiepileptic medications were taken by the IS patients according to their own conditions such as topiramate, oxcarbazepine, levetiracetam, and valproic acid. All IS patients underwent a comprehensive clinical evaluation and met the following inclusion criteria: (1) typical clinical symptoms and evident EEG findings of IS; (2) at least one seizure and consistent with the diagnosis of IS; (3) no other accompanying neurological disorders; and (4) without neurological or psychiatric disorders other than epilepsy. There was no significant difference between the two groups with respect to age (*t* = 0.467, *p* = 0.646) and gender (*t* = −0.956, *p* = 0.349) according to an independent samples *t*-test. All subjects were scanned by resting-state fMRI at the time of recruitment. Clinical details of all IS patients are given in Table 1. Written informed consent forms were obtained from the parents of all participants. The research protocol was approved by the Ethics Committee of Shenzhen Children’s Hospital, and the method was carried out according to the approved guidelines.

**Table 1:** **Characteristics of the IS patients**.

Patient	Sex	Age (months)	Onset time (months)	Type	Pathogeny	Seizure frequency (time/day)	Antiepileptic drugs
1	F	23	4	No lesion	No	3–4	LEV
2	M	83	24	No lesion	No	10–20	VPA TPM
3	F	3	3	Lesion	Brain cortex dysplasia	5–10	LEV OXC
4	M	8	0.5	Lesion	Temporal lobe cortex dysplasia	1–2	LEV VPA
5	M	18	6	Lesion	Cerebromalacia of right hemisphere	3–15	TPM
6	M	20	13	Lesion	Cerebromalacia of left hemisphere and right temporal lobe	10–20	VPA OXC
7	M	82	24	No lesion	Bilateral gray matter heterotopia	1–2^a^	LEV TPM
8	M	80	6	Lesion	Cerebromalacia of left hemisphere	3–4	CBZ VPA
9	F	13	5	No lesion	Bilateral parietal-occipital cortex dysplasia	7–10	TPM LEV
10	M	71	3	No lesion	Signal of right anterior cingulate cortex abnormal	1–2	TPM LEV
11	M	42	9	Lesion	Signal of right mesial temporal lobe abnormal	1–2	LEV OXC
12	M	5	5	No lesion	Bilateral pachygyria deformity	1^b^	TPM OXC
13	M	7	7	Lesion	Right temporal lobe lesion	1–3^c^	LEV

*^a^Time/year*.

*^b^Time/month*.

*^c^Time/week*.

*LEV, levetiracetam; VPA, valproic acid; TPM, topiramate; OXC, oxcarbazepine; CBZ, carbamazepine*.

### Image Acquisition

Functional MRI data were acquired on a 3-T scanner (MAGNETOM Trio Tim, Siemens, Germany) using an eight-channel whole-head coil at Shenzhen Children’s Hospital, Guangdong, China. The following parameters were used: TR/TE = 2,000/30 ms, matrix = 94 × 94, flip angle = 90°, FOV = 220 mm × 220 mm, slice thickness = 3 mm, 36 interleaved axial slices, and 130 volumes. During the resting-state recordings, all participants under the age of 4 years were sedated with 10% chloral hydrate. Others were instructed to relax with their eyes closed, keep their head still without falling asleep, and think of nothing. To avoid falling asleep, we performed a relatively short scan, observed throughout the whole scanning process, and asked their conditions after that. To limit head translation movement and rotation, foam cushions were used for each subject.

### Data Processing

Spatial preprocessing of fMRI data was performed using DPARSF software,^1^ which is based on the Statistical Parametric Mapping (SPM8)^2^ and REST^3^ package. To limit head translation and rotation, subjects with head motion more than 1.5 mm of maximal translation or 1.5°mm of maximal rotation throughout the course of scanning were excluded before from groups to obtain more stable data. To achieve a magnetized equilibrium, the first 10 volumes were removed for MRI signal. We followed the preprocessing steps according to a previous study, which encompassed slice timing, realignment, normalization, and spatial smoothing (Gaussian kernel, FWHM = 6 mm) (25). The fMRI data were then temporally filtered (band pass, 0.01–0.08 Hz) to reduce the very low-frequency drift and high-frequency respiratory and cardiac noises. For FC, spherical seed ROI with radius 6 mm was placed in posterior cingulate cortex (PCC), centered at MNI coordinates x = 3, y = −57, and z = 26. We chose PCC as our ROI to obtain DMN spatial pattern because it robustly elicits DMN maps and is not lateralized. Furthermore, PCC is considered as the only node in the default network that directly interacts with almost all other nodes according to Fransson and Marrelec (26). We obtained ALFF through calculating the square root of the power spectrum in the frequency range of 0.01–0.08 Hz (19).

### Statistical Analysis

Statistical analysis was performed using SPM8 software. As for FC, to obtain the distribution of spatial connectivity between the seed PCC and other areas in DMN for further analysis, one-sample *t*-test was performed to generate the *t* maps at the threshold of *p* < 0.05, corrected with false discovery rate (FDR) criterion (27). We saved the *t* maps obtained from the one-sample *t*-test of healthy controls whose regions were in showed active states as DMN mask for further intergroup comparison. Subsequently, two-sample *t*-test was compared under the mask to investigate the intergroup connectivity differences in DMN. The threshold was set at *p* < 0.05, corrected with the FDR criterion. The cluster was corrected to >10 adjacent voxels. As for ALFF, to find out which regions showed higher ALFF than global mean, we performed one-sample *t*-test against 1 (the mean value within the mask). After we saved the *t* maps (*p* < 0.05, FDR criterion) obtained from the one-sample *t*-test of healthy controls whose brain network showed higher ALFF than global mean as DMN mask, two-sample *t*-test was performed to observe the differences of ALFF between the two groups. We set the significance level at *p* < 0.05, corrected with FDR criterion. The cluster was corrected to >10 adjacent voxels.

## Results

### Between-Group FC Analysis of the DMN Connectivity

**Figure 1:**
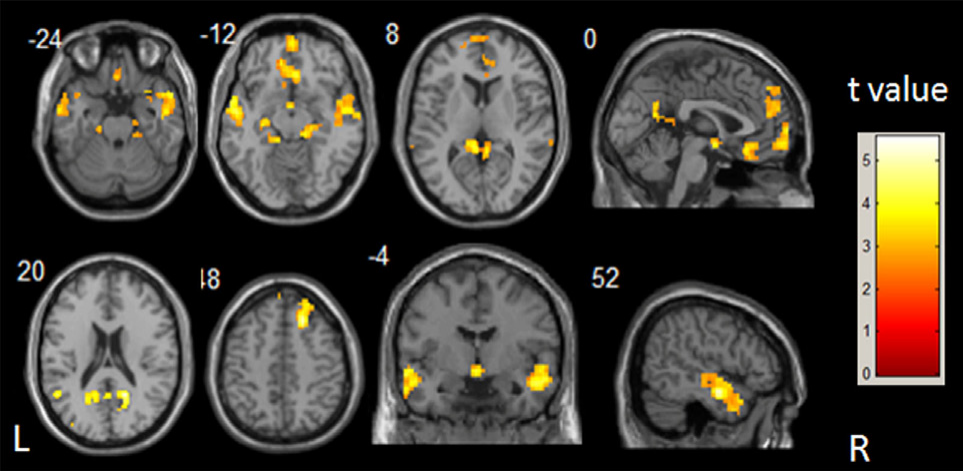
***t*-Statistical different maps between the infantile spasms patients and healthy controls of functional connectivity value (two-sample *t*-test; *p* < 0.05, false discovery rate correction)**. The bar to the right indicates the *t* score level. L, left; R, right.

The between-group differences in DMN, which was defined by the FC with seed in the PCC, were achieved through two-sample *t*-test (shown in Figure 1; Table 2). Compared with healthy control subjects, there was no increase in FC in the patients with IS. However, decreased connectivity was found at bilateral superior and middle temporal gyri, bilateral medial superior frontal gyri, bilateral fusiform gyri, lingual gyri, and parahippocampus in IS patients. Right precuneus, bilateral hippocampus, and cingulate gyri also showed a significantly decreased FC in the IS group.

**Table 2:** **Brain regions showing abnormal functional connectivity in IS patients**.

Brain region	MNI coordinates			*t* Value	Voxel number
	*X*	*Y*	*Z*		
Patients < controls					
Temporal_Mid_R	48	−9	−18	5.54	443
Temporal_Pole_Sup_R	30	15	−27	4.19	
Temporal_Pole_Mid_R	57	12	−27	4.10	
Frontal_Sup_R	21	27	48	5.24	206
Temporal_Mid_L	−54	−9	−15	4.66	221
Temporal_Sup_L	−60	0	−12	4.25	
Anterior Cingulate R	9	24	−6	4.54	174
Frontal_Med_Orb_L	−9	39	−12	4.02	
Rectus_R	3	30	−15	4.01	
Lingual_R	6	−42	0	4.52	324
Cingulate_Post_L	−9	−48	21	4.02	
Fusiform_R	21	−30	−18	4.23	94
Parahippocampa_R	15	−30	−12	3.87	
Hippocampus_R	30	−21	−15	3.06	
Hippocampus_L	−27	−21	−9	4.06	50
Parahippocampal_L	−18	−21	−21	3.33	
Frontal_Sup_Medial_L	0	66	3	4.04	118
Frontal_Med_Orb_R	3	60	−9	4.04	
Cingulum_Ant_R	6	48	21	3.94	251
Frontal_Sup_Medial_L	−3	57	24	3.85	
Frontal_Sup_Medial_R	6	57	39	3.77	
Corpus Callosum R	18	−39	21	3.66	46
Precuneus_R	15	−45	30	3.36	
Fusiform_L	−15	−36	−15	3.61	48
Lingual_L	−12	−33	−6	3.35	
Temporal_Pole_Sup_L	−42	21	−30	3.40	17
Temporal_Pole_Mid_L	−39	12	−27	3.16	
Temporal_Mid_L	−66	−36	3	2.79	10
Temporal_Sup_R	69	−36	12	2.78	10

*MNI, Montreal Neurological Institute; the MNI coordinates and t value for the local maxima of the centers of the voxel clusters; L, left; R, right*.

### Between-Group ALFF Analysis of the DMN

**Figure 2:**
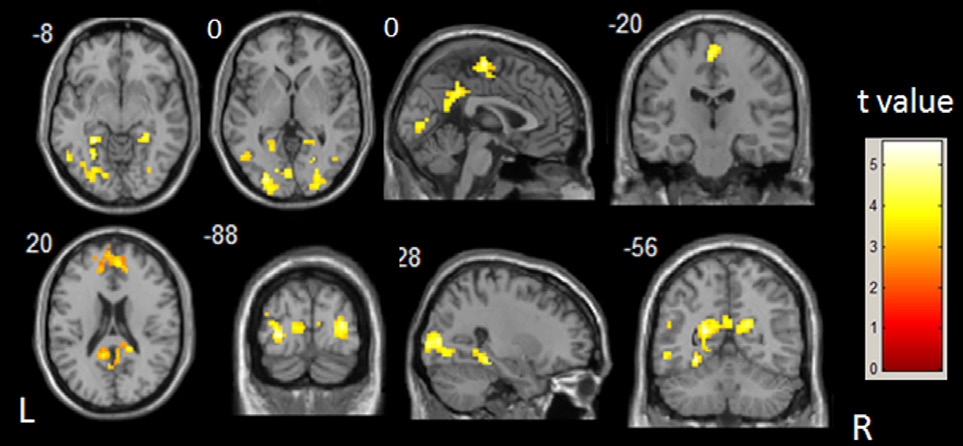
***t*-Statistical different maps between the infantile spasms patients and healthy controls of amplitude of low-frequency fluctuation value (two-sample *t*-test; *p* < 0.05, false discovery rate correction)**. The bar to the right indicates the *t* score level. L, left; R, right.

**Figure 3:**
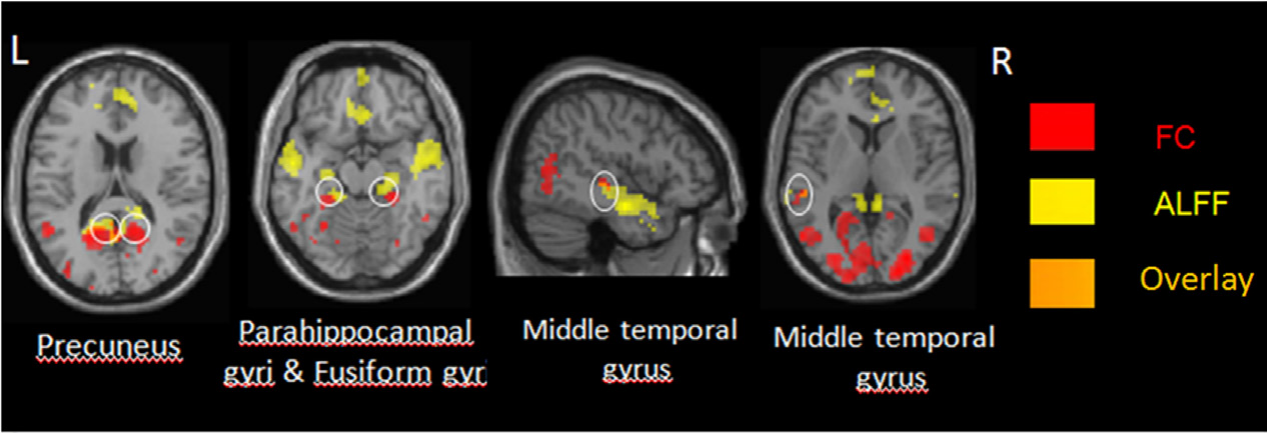
**The regions shown in Figures 1 and 2 overlap**. FC, functional connectivity; L, left; R, right.

The between-group differences in DMN, which was defined by the increased ALFF in healthy controls, were obtained through two-sample *t*-test (shown in Figure 2; Table 3). Compared with healthy control subjects, significantly decreased ALFF was found at bilateral inferior and middle temporal gyri, bilateral middle occipital gyri, parahippocampal gyri, and lingual gyri in IS group. Some other regions, such as precuneus, paracentral lobule, supplementary motor area, left angular gyrus, left fusiform gyrus, and middle cingulate gyrus, also showed a significantly decreased ALFF in the IS group. There was no significant increase in ALFF in the patients with IS. The commonly changed regions include bilateral middle temporal gyri, bilateral parahippocampal gyri, bilateral precuneus, and bilateral fusiform gyri after overlaying the images shown in Figures 1 and 2 (Figure 3).

**Table 3:** **Brain regions showing abnormal ALFF in IS patients**.

Brain region	MNI coordinates			*t* Value	Voxel number
	*X*	*Y*	*Z*		
Patients < controls					
Middle occipital gyrus	30	−84	6	5.17	152
Occipital_Mid_R	24	−93	6	4.87	
Lingual_R	24	−69	−3	4.23	
Temporal lobe	−21	−60	15	5.04	253
ParaHippocampal_L	−27	−42	−9	4.80	
Cingulum_Mid_L	0	−45	36	4.23	
Occipital_Mid_L	−24	−87	0	4.81	287
Fusiform_L	−27	−57	−12	4.59	
Lingual_L	−3	−81	0	4.21	
Precunneus_R	18	−54	21	4.67	66
Paracentral_Lobule_L	0	−18	63	4.59	65
Supp_Motor_Area_L	0	−12	51	3.57	
ParaHippocampal_R	27	−36	−12	4.43	50
Lingual_R	18	−48	−3	3.51	
Temporal_Mid_L	−51	−54	18	4.23	22
Temporal_Mid_L	−54	−66	9	4.12	61
Temporal_Inf_L	−54	−60	−9	3.87	
Temporal_Mid_L	−48	−63	0	3.45	
Angular_L	−42	−69	48	4.11	62
Occipital_Mid_L	−33	−81	36	4.01	
Occipital_Mid_L	−42	−72	27	3.74	
Temporal_Mid_R	48	−66	9	3.87	40
Temporal_Inf_R	45	−66	−6	3.78	
Postcentral_L	−45	−9	36	3.63	17

*ALFF, amplitude of low-frequency fluctuation; MNI, Montreal Neurological Institute; the MNI coordinates and t value for the local maxima of the centers of the voxel clusters; L, left; R, right*.

## Discussion

This is the first study to explore the alterations of brain activity in DMN in IS patients during resting state through FC and ALFF analyses. In FC analysis, compared with the healthy subjects, the correlation of the fMRI BOLD time series between PCC seed and DMN regions decreased in IS patients, such as bilateral temporal cortex, medial prefrontal cortex, and lingual gyri. In ALFF analysis, compared with healthy children, IS patients also showed decreased ALFF bilateral temporal cortex, occipital lobe cortex, and precuneus. In addition, coherent decreased regions among both methods were mainly located in the posterior of DMN cortical regions, and they were generally symmetrical. The main regions include bilateral middle temporal gyri, bilateral parahippocampal gyri, bilateral precuneus, and bilateral fusiform gyri (Figure 3). These commonly involved regions by both methods might reflect reduced DMN integration in IS patients.

### Decreased DMN FC in IS Group

Functional connectivity analysis as an efficient method has been widely used to identify spatial patterns of spontaneous coherent BOLD activity (28). In this study, we chose PCC as seed because it is typically used to obtain DMN spatial pattern in previous studies (29, 30). After FC analysis with the seed PCC, the spatial distribution of DMN regions from the control group was consistent with previous studies (18). This implied that the brain regions achieved through FC analysis with the seed PCC displayed coherent low-frequency BOLD fluctuations and indeed represented default mode.

In our study, correlation of the BOLD time series in DMN regions with the PCC seed decreased in IS group, which reflected decreased DMN functional integration. The result catered to many previous studies that the functional alterations of DMN regions are associated with the epileptic disease. A previous resting-state fMRI study found that FC between the lateral frontal node and lateral parietal node within DMN, which was significantly decreased in patients, had significant negative correlation with epilepsy duration (18). This result showed that the integration of DMN was interrupted with seizure. Another study on pediatric epilepsy also showed that reduced DMN connectivity was found in PCC, bilateral lateral parietal cortex, and anterior and midcingulate compared with controls (31). The evidence above supports the viewpoint that the alteration of DMN signals is related with the after-effects of seizure such as the functional disruption and reorganization in interictal durations. Different types of epilepsy also show different aberrant regions in DMN. The aberrant DMN regions with disturbed FC are likely to become one feature for neuroimage evidence in IS patients.

Abnormally interictal epileptic discharges (IEDs) might give rise to decreased FC and the long-term cognitive impairment in IS patients. FC is considered as temporal dependency of neuronal activation patterns of anatomically separated brain regions (32). A study using magnetic resonance encephalography has detected negative BOLD responses related to focal IED during focal IED occurrence in temporal epileptic patients, and it often occurred in DMN regions (33). This study showed that IED has a direct effect on the BOLD signal in DMN regions. In addition, a recent study on childhood absence epilepsy revealed that decreased FC was found within DMN during interictal generalized spike-wave discharges through EEG-fMRI (34). The above evidence supports that abnormal IEDs significantly contribute to the disruption of FC in epilepsy patients. IS patients are usually accompanied with asynchronous large amplitude slow waves mixed with single, multifocal spikes and sharp waves followed by attenuation. In our study, decreased FC might be the result of aberrant epileptic discharges. Further study with EEG during epileptic discharges will be performed in IS patients.

Functional connectivity reflects the level of functional communication through measuring the neuronal coactivation between regions. In the present study, we chose PCC as the seed that might affect our results. PCC is considered as the core of the DMN, and the posterior DMN regions are larger than the anterior ones. So it might be more sensitive to the effect of IS. Therefore, we combined ALFF to check the altered BOLD signal in each DMN region.

### Decreased DMN ALFF in IS

In this study, compared with control group, decreased ALFF was mainly located in the main area of DMN such as occipital cortex and prefrontal cortex. Previous studies in monkeys in simultaneous intracortical electrophysiology and imaging experiments have showed that a spatially located increase in the BOLD contrast directly and monotonically reflects an increase in neural activities (35). So these decreased regions in our study in DMN might reflect that local neuronal activities in DMN of IS patients were disrupted. The results were similar with a previous study on idiopathic complex partial seizures epilepsy of teenagers. In this study, the patients group also showed a decrease of ALFF in DMN (36). Another fMRI study on medial temporal lobe epilepsy also documented that decreased ALFFs were mostly located in the regions of DMN (24). A spontaneous abnormal discharge of the regions associated with epilepsy might lead to the disruption of neuronal activities when produced and spreaded. In addition, taking antiepilepsy drugs for long time might affect the functional alteration in IS patient.

### The Coherent Altered Areas in DMN in IS Patients with the Two Methods

In this study, the two analytical methods—FC and ALFF—were employed to explore the changes of DMN in IS patients. The commonly decreased regions in DMN obtained through the two methods help to confirm the disrupted integrity in DMN. The consistent altered regions encompass bilateral middle temporal gyri, bilateral parahippocampal gyri, bilateral precuneus, and bilateral fusiform gyrus. These regions are generally symmetrical. These consistently altered areas in DMN in IS patients might contribute to the functional interruption and reorganization.

In the present study, the commonly altered regions in DMN showed aberrant FC and ALFF signals compared with that in healthy controls. This result indicates that local neuronal activities of these regions are interrupted in IS patients. A recent study with event-related potentials design indicate that there might be a failure of temporal lobe maturation during infancy in IS patients (37). Another study in IS patients also showed that the white matter development in temporal lobe is impaired through tract-based statistics analysis (38). Combining with those previous studies, our results indicated that the functional alteration might relate to interrupted structure in IS patients. Significantly decreased connectivity is also found in precuneus during interictal generalized spike-wave discharges on childhood absence epileptic patients (34). We speculate that the neuronal disruption in precuneus in our study would reflect the effects of epilepsy on patient’s brain function. Parahippocampal gyrus is confirmed that it links the default mode cortical network with the medical temporal lobe memory system according to a recent study with fMRI (39). The neuronal disruption in parahippocampal gyrus in our study may relate to the mental retardation or regression happened in IS patients. Further study about the relations between altered regions and behavioral data will be performed.

### Limitations

The current study has several limitations. First, the sample sizes were not large enough, and further researches with larger sizes are needed to perform the correlation with the behavioral data. Second, simultaneous EEG during the MRI scanning was not collected. The current results could not completely confirm epilepsy discharges during MRI scanning. Third, the antiepileptic medications were taken by the IS patients without artificial control, which may disturb the results. In our study, one or two antiepileptic medications were taken by the IS patients among topiramate, lamotrigine, levetiracetam, and valproic acid. They can lead to some side effects such as tolerance, irritability, hepatotoxicity, and potential impaired cognition and learning in the process of pharmacotherapy in IS patients (40, 41). In the future, the functional alteration from different kinds of medications to IS patients needs to be explored with more data-related study.

## Conclusion

The present study demonstrates that the FC and ALFF in DMN are changed in IS patients. These alterations reflect the neuronal functional impairment. The consistent altered areas are mainly located in bilateral middle temporal gyri, bilateral lingual gyri, bilateral parahippocampal gyri, right precuneus, and left fusiform gyrus. The alteration in most of these regions has been showed to be significantly related to IED caused by abnormal epileptiform activity. They may serve as the indicators to reflect the developmental phase of IS patients.

## Ethics Statement

This study was carried out in accordance with the recommendations of approved guidelines of the Ethics Committee of Shenzhen Children’s Hospital with written informed consent from the parents of all subjects. All subjects gave written informed consent in accordance with the Declaration of Helsinki. The protocol was approved by the Ethics Committee of Shenzhen Children’s Hospital.

## Author Contributions

Conceived and designed the experiments: WH. Performed the experiments: YW. Analyzed the data: YW and YL. Contributed reagents/materials/analysis tools: HW. Responsible for patient management and conceptualized the study: YC. Wrote and revised the paper: YW and YL.

## Conflict of Interest Statement

The authors declare that the research was conducted in the absence of any commercial or financial relationships that could be construed as a potential conflict of interest.

## Footnotes

^1^http://www.rfmri.org/dparsf_v2_2.

^2^http://www.fil.ion.ucl.ac.uk/spm/.

^3^http://www.restfmri.net.
